# EQ-5D-Y-5L as a patient-reported outcome measure in psychiatric inpatient care for children and adolescents – a cross-sectional study

**DOI:** 10.1186/s12955-020-01366-4

**Published:** 2020-06-03

**Authors:** Mimmi Åström, Sonja Krig, Sara Ryding, Neil Cleland, Ola Rolfson, Kristina Burström

**Affiliations:** 1 Health Outcomes and Economic Evaluation Research Group, Stockholm Centre for Healthcare Ethics, Department of Learning, Informatics, Management and Ethics, Karolinska Institutet, Tomtebodavägen 18 A, SE-171 77 Stockholm, Sweden; 2 Equity and Health Policy Research Group, Department of Global Public Health, Karolinska Institutet, Stockholm, Sweden; 3 Health Care Services, Region Stockholm, Stockholm, Sweden; 4 Child and Adolescents Psychiatric Clinic, Region Stockholm, Stockholm, Sweden; 5 Department of Clinical Science and Education, Sodersjukhuset, Karolinska Institutet, Stockholm, Sweden; 6 Department of Orthopaedics, Institute of Clinical Sciences, the Sahlgrenska Academy, University of Gothenburg, Gothenburg, Sweden

**Keywords:** Adolescents, EQ-5D-Y-5L, Feasibility, Health-related quality of life (HRQoL), Patient-reported outcome measure, Psychiatric disorders, Psychometric properties, Strength and difficulties questionnaire (SDQ), Self-rated health, Validity

## Abstract

**Background:**

Psychiatric disorders have a major individual and societal impact. Until now, the association between health-related quality of life and physical disorders has been far more investigated than the association with psychiatric disorders. Patient-reported outcome measures makes it possible to capture the patient perspective to improve treatments and evaluate treatment outcomes. The aim of this study is to measure health-related quality of life with the EQ-5D-Y-5L among patients in child and adolescent psychiatric inpatient care and to test the instrument’s psychometric properties in terms of feasibility and construct validity.

**Methods:**

Data were collected at the child and adolescent psychiatric inpatient facility in Region Stockholm. A questionnaire including the EQ-5D-Y-5L instrument, the Strengths and Difficulties Questionnaire with an impact supplement and a self-rated health question, was administered for self-completion using paper and pencil, with an interviewer present. The Chi-square test was used to investigate differences in proportion of reported problems in the EQ-5D-Y-5L dimensions and the Mann-Whitney U test was used for differences in mean EQ VAS scores. Feasibility was assessed by investigating proportion of missing and ambiguous answers and Spearman’s and Pearson’s correlation were used to examine construct validity.

**Results:**

In total 52 adolescents participated in the study and the majority were girls. The most common diagnosis at admission was depressive episode/recurrent depressive disorder. All participants reported problems on at least one dimension. Most problems were reported in the dimension ‘feeling worried, sad or unhappy’, where 64% reported severe or extreme problems. Mean EQ VAS score was 29.2. Feasibility was supported and construct validity indicated as some of the hypothesised correlations between the EQ-5D-Y-5 L and the Strengths and Difficulties Questionnaire were found, however, for ‘doing usual activities’ and ‘having pain or discomfort’ the correlations were weaker than hypothesised.

**Conclusions:**

This is the first study where the newly developed EQ-5D-Y-5L instrument has been used in psychiatric inpatient care for youth. Participants reported problems in all severity levels in most of the EQ-5D-Y-5L dimensions; mean EQ VAS score was considerably low. Feasibility of the EQ-5D-Y-5L was supported, however other psychometric properties need to be further tested in a larger sample.

## Background

Psychiatric disorders have a major individual and societal impact. Beyond the significant burden of the disease on individuals’ health status and the burden on their families, psychiatric disorders are highly costly both for the individual and the society causing sickness absence, unemployment, poverty and premature death [1]. The World Health Organization describes psychiatric disorders as “a broad range of problems, generally characterized by some combination of abnormal thoughts, emotions, behaviour and relationships with others” [2]. Among children and adolescents, the prevalence of psychiatric disorders is 10–20%, and it is the leading cause of disability in this age group [3]. In Sweden, the prevalence, defined as one care episode within inpatient care or specialist outpatient care or at least one collection of psychotropic drugs, is around 10% [4].

Psychiatric disorders often emerge in children and adolescents during a period in life characterized by pressuring life events such as detaching from parents, finishing school and first experience with alcohol and drugs. These events may trigger or intensify psychiatric disorders, which might have been latent but affected quality of life for a long period of time [5]. Earlier studies show that children and adolescents with psychiatric disorders report substantially worse Health-Related Quality of Life (HRQoL) than the general population [6]. Among adults, generic HRQoL instruments have been shown to capture the impact on health status for some common psychiatric disorders [7–9]. However, the pattern of psychiatric disorders among youth might differ from adults, and as youth are going through phases of physical, emotional, behavioural and cognitive changes, it is important to test generic HRQoL instruments within this specific age group [5]. To use a generic HRQoL instrument in a specific patient group enables comparisons of HRQoL across disease groups [10]. Further, it is also important to decision makers in resource allocation, as improvements in health from different interventions are to be compared [11].

Using an instrument in a population or a context where it has not been used before requires testing of its psychometric properties [12]. This can incorporate assessment of feasibility and validity. Feasibility refers to whether an instrument is acceptable for respondents [10] and validity is an assessment of to which degree the instrument measures the construct it is intended to measure [13].

The EQ-5D-Y (Youth) instrument is a generic Patient-Reported Outcome Measure, suitable for children from eight years old, covering five dimensions of health and a Visual Analogue Scale (EQ VAS). There are two versions of the instrument, the EQ-5D-Y-3L with three severity levels [14, 15], and the EQ-5D-Y-5L with five severity levels [16]. The latter was recently developed based on the same premises as for the adult five-level version i.e. to be a more sensitive instrument and to reduce ceiling effects [17]. Psychometric properties of the EQ-5D-Y-3L have been tested in earlier studies, for instance among children and adolescents in general populations [18–20] and in youth with cystic fibrosis [21], functional disability [22], asthma [23], acutely and chronically ill [24] and among those with kidney disease [25]. For example, to assess convergent validity, correlations between the EQ-5D-Y-3L and the Kidscreen-27 have been investigated. In the multinational study, among the Swedish sample of school children, the strongest correlation (r = − 0.49) was found between the EQ-5D-Y-3L dimension ‘feeling worried, sad or unhappy’ and the psychological well-being dimension of Kidscreen-27 [18]. Among children and adolescents with functional disabilities, the strongest correlation (r = 0.68) was found between the EQ VAS and the physical well-being dimension of Kidscreen-27 [22]. In both studies, the strongest correlations were in general found between the EQ-5D-Y-3L dimension ‘feeling worried, sad or unhappy’, EQ VAS and the Kidscreen-27 dimensions [18, 22]. Furthermore, known-groups validity for the EQ-5D-Y-3L has previously been indicated as respondents with more problems on the Strengths and Difficulties Questionnaire (SDQ) reported significantly more problems in all EQ-5D-Y-3L dimensions except for ‘looking after myself’ [18]. Feasibility of the EQ-5D-Y-3L has been supported in previous studies by valid responses provided by; 90% [18], 100% [19]; 100% [22]; 96% [23], of the respondents. The EQ-5D-Y-5L has been compared to the EQ-5D-Y-3L in terms of ceiling effects and reliability [26] and in terms of responsiveness [27] among young scoliosis patients.

The aim of this study is to measure HRQoL with the EQ-5D-Y-5L among patients in child and adolescent psychiatric inpatient care and to test the instrument’s psychometric properties in terms of feasibility and construct validity.

## Methods

### Setting

Psychiatric specialist care in Sweden for children and adolescents younger than 18 years old is divided between local outpatient care facilities and inpatient care facilities [28]. Inpatient care constitutes a relatively small part of the organization and in 2014, around 400 children and adolescents with severe psychiatric disorders were treated within inpatient care in Region Stockholm. The most common diagnoses were severe depression, suicidal thoughts and attempts, severe eating disorders and psychotic or bipolar disorders [29]. Treatment duration for patients at the inpatient facility varies between one day and three weeks, however around 60% of the patients has a treatment period of one to two days [30].

In the present study, data were collected at the child and adolescent psychiatric inpatient facility in Region Stockholm. At the emergency care unit, data collection took place after the patient had stayed overnight for observation, and at the wards for inpatient care, between one and six days after admission depending on the health condition of the patient.

### Data collection and study participants

Healthcare personnel assessed the condition of each patient before the second author (SK), who is a registered nurse, informed the patients about the study and asked if they wanted to participate. Beside healthcare personnel’s assessment of the health condition of each patient, inclusion criteria for participation were being aged eight years and above, knowledge in the Swedish language, patients who had stayed overnight at the emergency care unit or who were admitted to one of the inpatient care wards. The recruitment of participants was based on convenience sampling, where those who fulfilled the inclusion criteria were asked to participate in the study. Potential participants and their parents/guardians were given oral information and separate letters regarding the study and an informed consent form to sign, by both the participant and the parents/guardians. If an adolescent was seeking care without a parent/guardian present and was aged 15 or older, written informed consent was only obtained from the adolescent, which is in accordance to Swedish law.

### Mode of administration

The questionnaire was administered in a separate room, for self-completion using paper and pencil, with an interviewer present (SK). Background information in terms of age, sex, diagnosis was subsequently collected from the patients’ records.

### Instruments

The questionnaire consisted of the EQ-5D-Y-5L instrument, a question on self-rated health and the SDQ with an impact supplement. In addition, open-ended questions regarding the perception of the questionnaire were included which will be analysed and presented elsewhere.

#### EQ-5D-Y-5L

The EQ-5D-Y-5L is developed for self-completion by children from eight years old [14, 16]. The instrument covers five dimensions of health: ‘mobility’, ‘looking after myself’, ‘doing usual activities’, ‘having pain or discomfort’ and ‘feeling worried, sad or unhappy’. Each dimension has five severity levels: ‘no problems’ (level 1), ‘a little bit of problems’ (level 2), ‘some problems’ (level 3), ‘a lot of problems’ (level 4) and ‘cannot/extreme problems’ (level 5). By combining the dimensions and the severity levels a health profile or a health state can be derived, with each digit representing the severity level of each dimension. For example, the health state 11523 represents ‘no problems’ in the first two dimensions ‘mobility’ and ‘looking after myself’, ‘cannot’ in the third dimensions ‘doing usual activities’, ‘a little’ ‘pain or discomfort’ and ‘rather’ in the last dimension ‘feeling worried, sad or unhappy’. For the EQ VAS, the respondents are asked to rate their health between 100 (the best health you can imagine) and 0 (the worst health you can imagine). The EQ-5D-Y-5L has a recall period of today.

#### Strengths and difficulties questionnaire (SDQ)

The SDQ instrument is developed for clinicians, educators and researchers to provide insight into children and adolescents’ behaviours, emotions and relationships [31]. SDQ has shown convergent and discriminant validity as well as acceptable test-retest reliability when tested among Swedish adolescents [32]. In Region Stockholm the SDQ is used within several psychiatric outpatient care facilities for children and adolescents with the main purpose to evaluate treatment effects [33]. The instrument is one of several instruments, recommended for assessment and treatment in psychiatric care for children and adolescents [34]. The instrument covers five scales: ‘conduct problems’, ‘emotional symptoms’, ‘peer relationship problems’, ‘hyperactivity/inattention’ and ‘prosocial behaviour’. Each scale contains five items with three response options: ‘not true’, ‘somewhat true’ and ‘certainly true’ [35]. In the present study, a self-completion version of the instrument developed for 11─17-year-olds, together with an impact supplement were used. The impact supplement is developed to enable investigation beyond symptoms and positive attributes [36]. Initially for the impact supplement, the respondent is asked whether he/she perceive difficulties with emotions, concentration, behaviour or ability to get on with other people. If the respondent answer ‘minor’, ‘definite’ or ‘severe’ difficulties, the respondent is asked to complete the remaining four questions regarding chronicity, impact and burden of difficulties [36]. The SDQ instrument has a recall period of six months. The SDQ total score was dichotomized with a cut-off point of 17 where participants with a score of 0–16 were considered having low risk, and 17–40 as having high risk of psychiatric problems [32].

#### Self-rated health

The self-rated health question is a valid measure of physical and mental health [37, 38], and is commonly used in health surveys among youth in Sweden [37]. The self-rated health question is included as the first question in the Kidscreen instrument, a validated and commonly used instrument to measure HRQoL in children and adolescents [39]. The self-rated health question was phrased: *‘How is your overall health? Is it: very good, good, neither good or bad, bad or very bad?’*. The response options were dichotomized into *very good/good/neither good or bad* and *bad/very bad*.

#### Diagnosis

Diagnosis was based on the primary diagnosis registered in the patient’s record on admission to the care facility. The diagnoses were grouped into units according to ICD-10 categories [40]. Primary diagnoses with less than five registrations were categorized as ‘other’.

### Ethical considerations

Ethical approval was granted by the Regional Ethical Review Board, Stockholm, Sweden (Dnr: 2017/2491–32; 2018/245–32).

### Data analyses

Proportion of adolescents reporting ‘no problems’, ‘a little bit of problems’, ‘some problems’, ‘a lot of problems’ and ‘cannot/extreme problems’ in each EQ-5D-Y-5L dimension was calculated and presented by age group, sex and diagnosis group. The sample was divided into the age groups 13–15 years and 16–17 years. Age was assessed as the exact age, in years, of the participant when completing the questionnaire. To test for statistically significant differences between groups in proportion of reported problems, the Chi-square test for trend was used and in some cases the severity levels were dichotomized into no problems and any problems before conducting the analysis [41]. Since EQ VAS scores were not normally distributed the Mann-Whitney U test was used to test for statistically significant differences in mean EQ VAS score between groups [41]. A 5% significance level was used for all analyses and which were performed in SPSS 23 [42].

#### Testing feasibility

Feasibility of the EQ-5D-Y-5L was assessed by examining the proportion of missing or ambiguous answers. Missing values were defined as a respondent not reporting their health on one of the dimensions or not providing a score on the EQ VAS; ambiguous answers were defined as a respondent choosing more than one severity level for a dimension or having an unclear mark [43]. Furthermore, feasibility was assessed by investigating the proportion of participants that self-completed the questionnaire and by time for completion.

#### Testing validity

Construct validity of the EQ-5D-Y-5L was assessed by convergent, discriminative and known-groups validity. Convergent validity was assessed by examining the relationship between EQ-5D-Y-5L dimensions and the EQ VAS score on the one hand and the SDQ scales and the self-rated health question that were hypothesised to be related (Table 1). Discriminant validity was assessed by examining if dimensions that were hypothesised to be unrelated also were unrelated. Spearman’s rank correlation and Pearson’s correlation were used to determine the strengths of correlations: negligible (0.00─0.09), weak (0.10─0.39), moderate (0.40─0.69), strong (0.70─0.89) and very strong (0.90─1.00) [44].

**Table 1:** Hypothesised correlations between the EQ-5D-Y-5L dimensions, EQ VAS score, SDQ and the Self-rated health question

Construct validity	EQ-5D-Y-5L	SDQ	Self-rated health	Hypothesised strengths of correlations^a^
Convergent	Mobility		Self-rated health	moderate
	Looking after myself	Hyperactivity/inattention		moderate
	Looking after myself	Emotional symptoms		moderate
	Doing usual activities	Impact supplement		moderate−strong
	Having pain or discomfort	Emotional symptoms		moderate−strong
	Having pain or discomfort	Conduct problems		moderate−strong
	Having pain or discomfort	SDQ total score		moderate
	Feeling worried, sad or unhappy	Emotional symptoms		moderate−strong
	Feeling worried, sad or unhappy	SDQ total score		moderate
	Feeling worried, sad or unhappy	SDQ impact supplement		moderate
	EQ VAS		Self-rated health	moderate−strong
	EQ VAS	SDQ total score		moderate−strong
	EQ VAS	SDQ impact supplement		moderate−strong
Discriminative	Mobility	All SDQ scales		negligible
	Mobility	SDQ total score		negligible
	Mobility	SDQ impact supplement		negligible
	All EQ-5D-Y-5 L dimensions	Prosocial behaviour		negligible−weak
	EQ VAS	Prosocial behaviour		negligible−weak

^a^Peasgood T, Bhardwaj A, Biggs K, Brazier JE, Coghill D, Cooper CL, et al. The impact of ADHD on the health and well-being of ADHD children and their siblings. Eur Child Adolesc Psychiatry. 2016;25(11):1217–31 [45]

The EQ-5D-Y-5L dimension ‘looking after myself’ was hypothesised to correlate with the SDQ scales ‘hyperactivity/inattention’ and ‘emotional symptoms’, as children and adolescents with hyperactivity previously have reported more problems in this dimension compared to the general population [45]. The dimension ‘doing usual activities’ was expected to correlate with the SDQ impact supplement as they cover similar aspects. The dimension ‘having pain or discomfort’ was expected to correlate with the SDQ scales ‘emotional symptoms’ and ‘conduct problems’, as it has previously been shown that children and adolescents with emotional and behavioural disorders have reported somatic symptoms [45]. The dimension ‘feeling worried, sad or unhappy’ was expected to correlate with the scale ‘emotional symptoms’, SDQ total score and SDQ impact supplement. The EQ VAS score was expected to correlate with severity levels of the self-rated health question, the SDQ total score and SDQ impact supplement. Negligible or weak correlations i.e. discriminant validity, were hypothesised between the dimension ‘mobility’ and all the SDQ scales, the SDQ total score and the SDQ impact supplement and the severity levels of the self-rated health question. Negligible or weak correlations were also expected between all the EQ-5D-Y-5L dimensions and the SDQ scale ‘prosocial behaviour’ (Table 1).

Known-groups validity was assessed by examining if, as expected, participants with worse self-rated health reported more problems in the EQ-5D-Y-5L dimensions and had lower mean EQ VAS score than those with better self-rated health. Known-groups validity was also assessed by examining if, as expected, participants with higher SDQ total score reported more problems in the EQ-5D-Y-5L dimensions and had lower mean EQ VAS score than those with a lower SDQ total score.

### Characteristics of study participants

In total, 52 patients participated in the study and completed the questionnaire. The participants were 13–17 years and the mean age was 15.4 years. A majority, 83% (*n* = 43), of the participants were girls. The most common diagnosis when admitted to the care facility was depressive episode/recurrent depressive disorder (29%, *n* = 15). More than half of the study participants, 54% (*n* = 28) had a psychiatric or behavioural comorbidity or a disorder of psychological development, and 73% (*n* = 38) self-reported that they had had their psychiatric problems for more than one year. Less than good self-rated health was reported by 96% (*n* = 50) and 67% (*n* = 35) reported their self-rated health as *bad* or *very bad.* None of the participants reported *very good* self-rated health (Table 2).

**Table 2:** Characteristics of study participants (*n* = 52)

Mean age	15.4 (1.4) Years (SD)	
	%	n
Age		
13 years	15	8
14 years	14	7
15 years	17	9
16 years	30	15
17 years	25	13
Sex		
Boys	17	9
Girls	83	43
Diagnostic group according to ICD-10^a^		
Bipolar affective disorder (F31)	12	6
Depressive episode/recurrent depressive disorder (F32–33)	30	15
Other anxiety disorder (F41)	25	13
Reaction to severe stress/ adjustment disorders (F43)	12	6
Other^b^	14	7
Observation for suspected mental and behavioural disorders (Z03.2)	10	5
Co-morbidity		
Yes	54	28
Duration of mental health problems		
< 1 month	0	0
1–5 months	12	6
6–12 months	6	3
> 1 year	73	38
Missing	10	5
Self-rated health		
Very good	0	0
Good	4	2
Neither good or bad	30	15
Bad	33	17
Very bad	35	18
Care setting for data collection		
Emergency department	73	38
Inpatient care wards	27	14

^a^Diagnosis when admitted

^b^Other = Mental and behavioural disorders due to multiple drug use and use of other psychoactive substances: harmful use (F19.1); unspecified nonorganic psychosis (F29.9); sleep disorder – unspecified (G47.9); eating disorder, unspecified (F50.9); problems in relationship with parents and in-laws (Z63.1)

^c^Self-reported in the SDQ questionnaire

## Results

All participants reported problems on at least one of the EQ-5D-Y-5L dimensions, i.e. no participant reported the health profile 11111. Among the 52 respondents, there were 48 unique health profiles and the most common profile was 11313 (*n* = 3), followed by the health profiles 11113 (*n* = 2) and 11435 (n = 2). Problems at the most severe level (level 5) for one or more dimensions were reported by 13 patients. Out of the 48 health profiles, 39 consisted of reported problems on the next to worst (level 4) or the worst (level 5) severity level.

### Prevalence of reported problems and mean EQ VAS score

In total, most problems were reported in the dimension ‘feeling worried, sad or unhappy’ where 87% reported level 3 or worse problems, followed by problems in the dimension ‘doing usual activities’ where 81% reported level 3 or worse. In the mood dimension 40% (*n* = 21) reported ‘really’ (level 4) and 23% (*n* = 12) reported ‘extremely’ (level 5) ‘worried, sad or unhappy’. Older participants, 16–17 years, had a higher prevalence of problems in all EQ-5D-Y-5L dimensions except ‘having pain or discomfort’ where the younger participants reported more problems. Mean EQ VAS score in the total sample was 29.2. The younger age group reported lower mean EQ VAS score (25.3) compared to the older age group (32.6). However, none of these differences between age groups were statistically significant, except for ‘having pain or discomfort’ when the response options were dichotomized (Table 3).

**Table 3:** Distribution (%,n) of reported problems in the EQ-5D-Y-5L dimensions, EQ VAS mean score (SD) and EQ VAS median, by age group

	Total		13–15 years		16–17 years		
EQ-5D-Y-5L dimensions	*n* = 52		*n* = 24		*n* = 28		
	%	n	%	n	%	n	*p*-value
Mobility (walking about)							
No problems	73	38	75	18	71	20	0.768^a^
A little bit of problems	21	11	17	4	25	7	
Some problems	4	2	4	1	4	1	
A lot of problems	2	1	4	1	0	0	
Cannot	0	0	0	0	0	0	
Looking after myself							
No problems	52	27	54	13	50	14	0.781^a^
A little bit of problems	37	19	29	7	43	12	
Some problems	12	6	17	4	7	2	
A lot of problems	0	0	0	0	0	0	
Cannot	0	0	0	0	0	0	
Doing usual activities							
No problems	12	6	13	3	11	3	0.812^a^
A little bit of problems	8	4	13	3	4	1	
Some problems	40	21	29	7	50	14	
A lot of problems	25	13	25	6	25	7	
Cannot	15	8	21	5	11	3	
Having pain or discomfort							
No pain or discomfort	27	14	13	3	39	11	0.308^a,b^
A little pain or discomfort	35	18	50	12	21	6	
Some pain or discomfort	21	11	21	5	21	6	
A lot of pain or discomfort	14	7	8	2	18	5	
Extreme pain or discomfort	4	2	8	2	0	0	
Feeling worried, sad or unhappy							
Not worried, sad or unhappy	6	3	8	2	4	1	0.969^a^
A little worried, sad or unhappy	8	4	8	2	7	2	
Rather worried, sad or unhappy	23	12	13	3	32	9	
Really worried, sad or unhappy	40	21	50	18	32	9	
Extremely worried, sad or unhappy	23	12	21	5	25	7	
	*n* = 51		*n* = 24		*n* = 27		
EQ VAS mean (SD)	29.2 (19.5)		25.3 (19.4)		32.6 (19.3)		0.16^c^
EQ VAS median	30		21		31		

^a^*p*-value by Chi-square test for trend

^b^*p*-value = 0.030, when dichotomized no pain or discomfort vs. any pain or discomfort

^c^*p*-value by Mann-Whitney U test

Both girls and boys reported most problems in the EQ-5D-Y-5L dimension ‘feeling worried, sad or unhappy’ followed by reported problems in the dimension ‘doing usual activities’. Girls reported a mean EQ VAS score of 28.1 and boys a mean EQ VAS score of 34.0 (results not shown).

Reported problems in the EQ-5D-Y-5L dimensions and EQ VAS mean and median score by diagnosis group are shown in Table 3. Participants in the group ‘other anxiety disorder’ and ‘other’ reported the lowest mean EQ VAS scores, 23.5 and 23.1, respectively (Table 4).

**Table 4:** Distribution (%, n) of reported problems in the EQ-5D-Y-5L dimensions, EQ VAS mean score (SD) and EQ VAS median score by diagnosis group

	Bipolar affective disorder (F31)		Depressive episode/recurrent depressive disorder (F32–33)		Other anxiety disorder (F41)		Reaction to severe stress/ adjustment disorders (F43)		Other^b^		Observation for suspected mental and behavioural disorders (Z03.2)	
	*n* = 6		*n* = 15		*n* = 13		*n* = 6		*n* = 7		*n* = 5	
EQ-5D-Y-5L dimensions	%	n	%	n	%	n	%	n	%	n	%	n
Mobility (walking about)												
No problems	83	5	67	10	54	7	83	5	86	6	100	5
A little bit of problems	0	0	33	5	31	4	17	1	14	1	0	0
Some problems	17	1	0	0	8	1	0	0	0	0	0	0
A lot of problems	0	0	0	0	8	1	0	0	0	0	0	0
Cannot	0	0	0	0	0	0	0	0	0	0	0	0
Looking after myself												
No problems	50	3	40	6	39	5	67	4	71	5	80	4
A little bit of problems	33	2	47	7	39	5	33	2	27	2	20	1
Some problems	17	1	13	2	23	3	0	0	0	0	0	0
A lot of problems	0	0	0	0	0	0	0	0	0	0	0	0
Cannot	0	0	0	0	0	0	0	0	0	0	0	0
Doing usual activities												
No problems	17	1	7	1	8	1	17	1	14	1	20	1
A little bit of problems	0	0	7	1	8	1	0	0	14	1	20	1
Some problems	67	4	33	5	46	6	50	3	29	2	20	1
A lot of problems	17	1	40	6	15	2	17	1	29	2	20	1
Cannot	0	0	13	2	23	3	17	1	14	1	20	1
Having pain or discomfort												
No pain or discomfort	67	4	27	4	8	1	33	2	14	1	40	2
A little pain or discomfort	17	1	33	5	54	7	50	3	14	1	20	1
Some pain or discomfort	0	0	33	5	8	1	17	1	29	2	40	2
A lot of pain or discomfort	17	1	7	1	23	3	0	0	29	2	0	0
Extreme pain or discomfort	0	0	0	0	8	1	0	0	14	1	0	0
Feeling worried, sad or unhappy												
Not worried, sad or unhappy	17	1	0	0	0	0	0	0	29	2	0	0
A little worried, sad or unhappy	0	0	7	1	15	2	0	0	14	1	0	0
Rather worried, sad or unhappy	33	2	0	0	15	2	50	3	29	2	60	3
Really worried, sad or unhappy	33	2	87	13	31	4	17	1	14	1	0	0
Extremely worried, sad or unhappy	17	1	7	1	39	5	33	2	14	1	40	2
EQ VAS mean (SD)	28.3 (27.7)		28.2 (14.4)		23.5 (15.8)^b^		42.5 (23.2)		23.1 (15.1)		39.0 (28.6)	
EQ VAS median	30		30		21		47.5		22		35	

^a^Other = Mental and behavioural disorders due to multiple drug use and use of other psychoactive substances: harmful use (F19.1); unspecified nonorganic psychosis (F29.9); sleep disorder – unspecified (G47.9); eating disorder, unspecified (F50.9); problems in relationship with parents and in-laws (Z63.1)

^b^*n* = 12

#### Feasibility

Feasibility was supported as there were no missing or ambiguous answers on the EQ-5D-Y-5L dimensions, and one missing value on the EQ VAS. Nearly all, but three, completed the questionnaire independently. All participants completed the EQ-5D-Y-5L in less than five minutes. Most participants, 62%, (*n* = 32) completed the questionnaire in the presence of the interviewer only, while 37% (*n* = 19) also had a parent present, and in one case a support person from social services was present during completion.

#### Construct validity

Moderate correlations were found between the EQ-5D-Y-5L dimension ‘feeling worried, sad or unhappy’ and the SDQ scale ‘emotional symptoms’ (*r* = 0.61; *p* < 0.001), the SDQ total score (*r* = 0.58; *p* < 0.001), and the SDQ impact supplement (*r* = 0.51; *p* < 0.001) (Table 5). Weak correlation was found between the dimension ‘doing usual activities’ and SDQ impact supplement (*r* = 0.38; *p* = 0.008). Weak correlations were also found between the dimension ‘having pain or discomfort’ and the SDQ total score (*r* = 0.33; *p* = 0.016) and the SDQ scales ‘emotional symptoms’ (*r* = 0.28; *p* = 0.042) and ‘conduct problems’ (*r* = 0.35; *p* = 0.012). The correlations were weak between the dimension ‘looking after myself’ and the SDQ scales ‘emotional symptoms’ (*r* = 0.22; *p* = 0.125) and ‘hyperactivity/inattention’ (*r* = 0.14; *p* = 0.318). The EQ VAS score correlated strongly with the severity levels of the self-rated health question (*r* = − 0.70; *p* < 0.001), and moderately with the SDQ total score (*r* = − 0.43; *p* = 0.002) and the SDQ impact supplement (*r* = − 0.45; *p* = 0.002) (Table 5).

**Table 5:** Spearman’s rank correlation between the EQ-5D-Y-5L, EQ VAS and Strengths and Difficulties Questionnaire (SDQ), Self-rated health

	Strengths and Difficulties Questionnaire (SDQ)							Self-rated health
	Emotional symptoms	Conduct problems	Hyperactivity/inattention	Peer problems	Prosocial behaviour	Total score	Impact supplement^c^	
EQ-5D-Y-5L dimensions	r_s_	r_s_	r_s_	r_s_	r_s_	r_s_	r_s_	r_s_
Mobility (walking about)	0.12	0.08	−0.04	0.24	−0.09	0.12	−0.02	0.04
Looking after myself	0.22	0.17	0.14	0.16	−0.02	0.24	0.13	0.12
Doing usual activities	0.20	0.06	0.04	0.15	−0.02	0.17	0.38**	0.30*
Having pain or discomfort	0.28*	0.35*	0.13	0.12	−0.06	0.33*	0.10	0.36**
Feeling worried, sad or unhappy	0.61**	0.23	0.34*	0.35*	0.12	0.58**	0.51**	0.57**
EQ VAS^a^	−0.36**	−0.21	−0.46**	−0.20	−0.04	−0.43**^b^	−0.45**	−0.70**

*p < 0.05, **p **< 0.01**

^a^*n* = 51

^b^ = Pearson correlation

^c^*n* = 47

The correlation between the self-rated health question and the EQ-5D-Y-5L dimension ‘feeling worried, sad or unhappy’ was strong (*r* = 0.57; *p* < 0.001), and moderate with the dimensions and ‘having pain or discomfort’ (*r* = 0.36; *p* = 0.009) and ‘doing usual activities’ (*r* = 0.30; *p* = 0.034) (Table 5).

Negligible or weak correlations were found between the EQ-5D-Y-5L dimension ‘mobility’ and all the SDQ scales, SDQ total score and SDQ impact supplement as well as with the severity levels of the self-rated health question. Furthermore, weak correlations were also found between all EQ-5D-Y-5L dimensions and the SDQ scale ‘prosocial behaviour’ (Table 5).

#### Known-groups validity

Known-groups validity was supported as patients who reported worse self-rated health also reported more problems in all the EQ-5D-Y-5L dimensions, however, statistical significant differences between groups were restricted to the dimensions ‘having pain or discomfort’ (*p* = 0.026) and ‘feeling worried, sad or unhappy’ (*p* = 0.005). Patients with worse self-rated health reported statistically significant lower mean EQ VAS score (20.8) compared to those with better self-rated health (45.8) (*p* < 0.001) (Table 6).

**Table 6:** Distribution (%, n) of reported problems in the EQ-5D-Y-5L dimensions, EQ VAS mean score (SD) and EQ VAS median score, by answer to the self-rated health question

	Self-rated health				
	Very good/good/neither good or bad		Bad/very bad		
EQ-5D-Y-5L dimensions	*n* = 17		*n* = 35		
	%	n	%	n	*p*-value
Mobility (walking about)					
No problems	77	13	71	25	0.958^a^
A little bit of problems	12	2	26	9	
Some problems	12	2	0	0	
A lot of problems	0	0	3	1	
Cannot	0	0	0	0	
Looking after myself					
No problems	59	10	49	17	0.954^a^
A little bit of problems	24	4	43	15	
Some problems	18	3	9	3	
A lot of problems	0	0	0	0	
Cannot	0	0	0	0	
Doing usual activities					
No problems	18	3	9	3	0.184^a^
A little bit of problems	0	0	11	4	
Some problems	59	10	31	11	
A lot of problems	18	3	29	10	
Cannot	6	1	20	7	
Having pain or discomfort					
No pain or discomfort	53	9	14	5	0.026^a^
A little pain or discomfort	24	4	40	14	
Some pain or discomfort	12	2	26	9	
A lot of pain or discomfort	12	2	14	5	
Extreme pain or discomfort	0	0	6	2	
Feeling worried, sad or unhappy					
Not worried, sad or unhappy	6	1	6	2	0.005^a^
A little worried, sad or unhappy	18	3	3	1	
Rather worried, sad or unhappy	47	8	11	4	
Really worried, sad or unhappy	24	4	49	17	
Extremely worried, sad or unhappy	6	1	31	11	
	*n* = 17		*n* = 34		
EQ VAS mean (SD)	45.8 (17.7)		20.8 (14.5)		0.000^b^
EQ VAS median	50		20		

^a^*p*-value by Chi-square test for trend

^b^*p*-value by Mann-Whitney U test

**Fig. 1:**
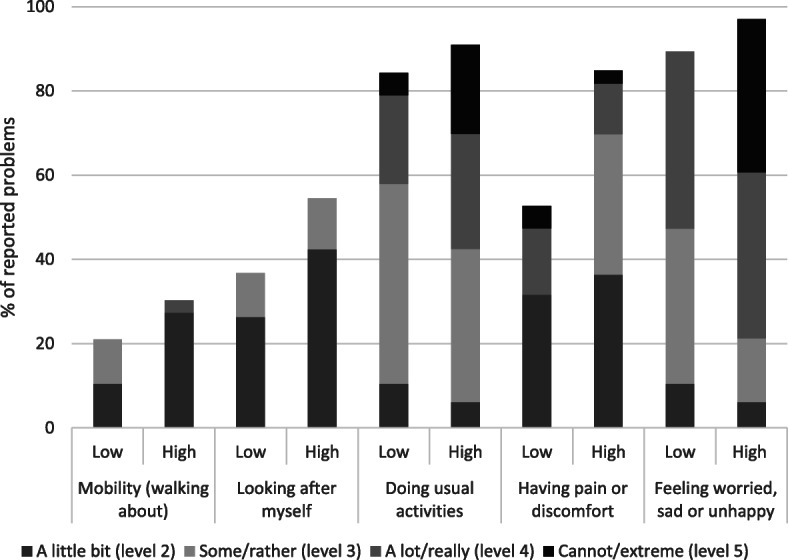
Difference in proportion of reported problems by SDQ total score

Participants with a SDQ total score of 17 and above, i.e. those who reported more problems on the SDQ, also reported more problems in all the EQ-5D-Y-5L dimensions (statistically significant for the dimension ‘feeling worried, sad or unhappy’, *p* = 0.005) (Fig. 1) and had statistically significant (*p* = 0.002) lower mean EQ VAS score (22.9) compared to those with SDQ total score less than 17 (39.7).

## Discussion

This study is, to the best of our knowledge the first study where the newly developed EQ-5D-Y-5L instrument has been used within psychiatric inpatient care for youth. Feasibility of the EQ-5D-Y-5L instrument was supported as few missing and ambiguous values were observed. Some of the hypothesised correlations between the EQ-5D-Y-5L, the SDQ and the self-rated health question were found, which might indicate construct validity, however further studies are needed.

As expected, most problems were reported in the EQ-5D-Y-5L dimension ‘feeling worried, sad or unhappy’. However, a large proportion of the patients also reported problems in the worst severity level (level 5) in the dimensions ‘doing usual activities’ and ‘having pain or discomfort’. These findings suggest that the EQ-5D-Y-5L, even though its generic nature, can capture decrements in health status beyond the mood dimension, among adolescents with psychiatric disorders. For most of the EQ-5D-Y-5L dimensions, the patients used all the severity levels to report their problems, this could be an indication that five severity levels are desirable when measuring HRQoL in this patient group. The proportion of reported problems in the present study was considerable high compared to reported problems in the general population of adolescents [46]. For e.g. in the dimension ‘doing usual activities’ 89% compared to 9% in the general population reported ‘any’ problems and in the dimension ‘feeling worried, sad or unhappy’ any problems were reported by 94% of the sample compared to 38% in the general population. Considering the healthcare setting in which the present study was conducted, these findings are not unexpected as this is one of the most vulnerable patient groups.

Adolescents in the present study reported remarkable low mean EQ VAS score (29.2) and median EQ VAS score (30) compared to what have been observed in earlier studies using the EQ-5D-Y-3L instrument [22, 23, 47]. Children and adolescents with functional disabilities have reported a mean EQ VAS score of 71.1 in Burström et al. [22] and a mean EQ VAS score between 76.9─88.1 in Domellöf et al. [48], whilst youth with asthma in a study by Bergfors et al. reported a mean EQ VAS score of 80.7 [23]. The median EQ VAS score in the present study is also notable low compared to hospitalized physically acutely ill children in Scott et al., who had a median EQ VAS score of 50 [24]. Considerable differences were observed when comparing the results from these adolescent patients to adolescents in the general population in Sweden in the study by Åström et al. [46] where the mean EQ VAS score was 75.4 (median 80). The low mean EQ VAS score in the present study is supported by a sub-group analysis showing a mean EQ VAS score of 58.9 among those adolescents in the general population who self-reported that they were often or always depressed [46]. Further investigation regarding what drives the low EQ VAS score is needed, however, it has been shown among adults that the mood dimension has a strong impact on the EQ VAS score when respondents value their own current health state [37].

Correlation indicating convergent validity was found between the dimension ‘feeling worried, sad or unhappy’ and the SDQ scale ‘emotional symptoms’, however this correlation was hypothesised to be strong but came out as moderate. Similar was shown for the dimension ‘looking after myself’ and the SDQ scales ‘hyperactivity/inattention’ and ‘emotional symptoms’ where moderate correlations were hypothesised but weak correlations were found. Between the dimension ‘doing usual activities’ and the SDQ impact supplement as well as between ‘having pain and discomfort’ and ‘emotional symptoms’ and ‘conduct problems’, the correlations were weak but hypothesised to be moderate or strong. The expected moderate to strong correlations between EQ VAS and the self-rated health question, the SDQ total score and the SDQ impact supplement were confirmed. There was no significant correlation between the dimension ‘looking after myself’ and any of the SDQ scales. In general, the hypothesised correlations were found, however, for the dimensions ‘doing usual activities’ and ‘having pain or discomfort’ the correlations were weaker than hypothesised. In this study, the guidelines for moderate and strong correlation were 0.4 and 0.7, respectively, whilst some other studies used guidelines with limits of 0.3 and 0.5, respectively [18, 22, 23].

Discriminant validity was indicated as the hypothesised negligible or weak correlations between the EQ-5D-Y-5L dimension ‘mobility’ and SDQ were found. Furthermore, as hypothesised we found negligible or weak correlations between EQ-5D-Y-5L and the SDQ scale ‘prosocial behaviour’. To assess construct validity by setting up hypothesis regarding the degree of correlations between the EQ-5D-Y-5L dimensions and dimensions in other instrument might be challenging, due to lack of literature to base these hypotheses on. The fact that several of the hypothesised correlations came out weaker than expected suggests that construct validity especially in terms of convergent validity of the EQ-5D-Y-5L needs to be further investigated in this patient group.

Known-groups validity was supported as patients with worse self-rated health and more problems on the SDQ, also reported more problems in all the EQ-5D-Y-5L dimensions and had lower mean EQ VAS score. The findings were similar when known-groups validity was previously investigated by comparing the EQ-5D-Y-3L to the SDQ [18].

This study has several limitations. The sample size was based on convenience sampling, where those who fulfilled the inclusion criteria and were assessed by healthcare personnel to manage to participate were asked to participate. In respect to the severity of the psychiatric illness among patients at this facility and their vulnerability, it was not possible to recruit more patients. Other methods such as factor analysis could have been an option to investigate construct validity with a larger sample. A suggestion for future research, to be able to recruit more participants in a systematic way, is to test the EQ-5D-Y-5L as a routine measurement in child and adolescents psychiatric inpatient care and to follow up health status among patients from admission to discharge. This approach would also enable to test other psychometric properties of the instrument e.g. test-retest reliability.

Furthermore, the timing of completion of the questionnaire could be questioned, as it differed between one to six days after admission. However, a vast majority in the sample answered the questionnaire within one or two days of admission. To complete the questionnaire at different points in time might affect the results, and a more systematic approach where all patients are asked to complete the questionnaire during the first day at the facility is suggested for future data collection. We have no data regarding how many who said no to participate in the study or the characteristics of those patients. Patients who were assessed by healthcare personnel to be too ill to participate in the study were excluded. This may have resulted in an underestimation of reported problems for the patient group. It is important to remember that potential participants were asked to participate in a research project, with the main aim to investigate a questionnaire, and data were not collected as a routine along healthcare delivery, this may have affected the willingness to participate. Few boys participated in the study, hence, it is not possible to analyse data divided by sex and no conclusions regarding differences between sexes can be drawn. However, considering both psychiatric in- and outpatient care for children and adolescents, there are more girls than boys being treated [29].

We had an opportunity to carry out the study and collect data with the already employed SDQ instrument in this psychiatric inpatient care clinic during an agreed time period in collaboration with the personnel. However, the differences in recall period between the EQ-5D-Y-5L which is ‘today’ and the SDQ which is ‘six months’ must be considered. It could be argued that a recall period of ‘today’ is suitable in the context of the present study, investigating HRQoL among youth in an acute care setting.

Health economists and policy makers favour generic HRQoL instruments to enable calculation of Quality-Adjusted Life Years (QALYs) for use in economic evaluation and resource allocation [49]. As for today, there is no value set for any of the EQ-5D-Y versions, however in the future, it is important to develop a value set for both the EQ-5D-Y-3L and EQ-5D-Y-5L to make it possible to use the instruments in economic evaluation of healthcare [18].

Conclusions based on the results from the present study should be drawn with caution, as this was a small sample. Furthermore, as this is a cross-sectional study, no conclusions regarding causality can be drawn and the psychometric properties need to be further investigated in a larger sample of study participants in this healthcare setting. Despite the limitations presented in the present study, the findings suggest that the EQ-5D-Y-5L can capture decrement in HRQoL in this patient group, however it is important to further test responsiveness of the instrument and to continuously to apply it in parallel with other instruments.

## Conclusions

This is the first study where the newly developed EQ-5D-Y-5L instrument has been used in psychiatric inpatient care for youth. Participants reported problems in all severity levels in most of the EQ-5D-Y-5L dimensions and the mean EQ VAS score was considerably low. Feasibility of the EQ-5D-Y-5L in this healthcare setting was supported but other psychometric properties need to be further tested in a larger sample. This study indicates initial support for the use of the EQ-5D-Y-5L as a patient-reported outcome measure among adolescents in psychiatric inpatient care for youth.

## Data Availability

Data sharing is not possible according to Swedish law.

## Abbreviations

EQ-5D-Y-5L: EuroQol 5 Dimensions Youth 5 Level
EQ VAS: EQ Visual Analogue Scale
HRQoL: Health-Related Quality of Life
SDQ: Strength and Difficulties Questionnaire
QALYs: Quality-Adjusted Life Years
